# Right Hemisphere Involvement in Non-Fluent Primary Progressive Aphasia

**DOI:** 10.1155/2007/423631

**Published:** 2008-03-05

**Authors:** Claudia Repetto, Rosa Manenti, Maria Cotelli, Marco Calabria, Orazio Zanetti, Barbara Borroni, Alessandro Padovani, Carlo Miniussi

**Affiliations:** ^1^IRCCS S. Giovanni di Dio FatebenefratelliBresciaItaly; ^2^Department of NeuroscienceVita Salute University and San Raffaele Scientific InstituteMilanItaly; ^3^Department of General PsychologyUniversity of PaduaItaly; ^4^Department of NeurologyUniversity of BresciaItaly; ^5^Department of Biomedical Sciences and BiotechnologiesUniversity of BresciaItaly

**Keywords:** Non-fluent primary progressive aphasia, language disorder, lateralization, frontotemporal dementia

## Abstract

We described a 56-years-old man with a diagnosis of “non-fluent primary progressive aphasia” (NfPPA). An accurate neuropsychological, neurological and neuroimaging evaluation was performed in order to assess clinical and behavioural features of the patient.

From a neuropsychological point of view, the patient showed a typical cognitive profile of subjects affected by NfPPA: a prominent language deficit, associated with impairments in several cognitive domains after three years from the onset of the symptomatology.

The most intriguing feature is that SPECT revealed hypoperfusion in the right frontal cortex, albeit the patient is right-handed.

This unexpected finding shows that NfPPA may arise not only from cortical abnormalities in the language-dominant left hemisphere, but also from right hemisphere involvement in a right hander (crossed aphasia).

## 1. Introduction

Primary Progressive Aphasia (PPA) is a neurodegenerative disease characterized by a) insidious onset, but progressive impairment of word finding, object naming, syntax, or word comprehension manifested during conversation or assessed with the use of standard neuropsychological tests of language; b) all major limitation in activities of daily living can be attributed to the language impairment for at least two years after onset; c) premorbid language function is known to be intact; d) prominent apathy, disinhibition, loss of memory of recent events, visuospatial impairment, visual-recognition deficits, and absence of sensory-motor dysfunction during the initial two years of illness, d) acalculia and ideomotor apraxia can be present even in the first two years of illness; e) other cognitive functions may be affected after the first two years of illness, but language remains the most impaired function throughout the course of the illness and deteriorates faster than other affected functions; f) specific cause of aphasia, such as stroke or tumor, as ascertained by neuroimaging, are absent [14,16].

PPA is observed in different forms: non-fluent PPA (NfPPA), characterized by effortful articulation, agrammatism, phonemic paraphasias, various degrees of anomia but relatively preserved comprehension; semantic dementia (SD), or fluent-PPA, in which prominent loss of the meaning of words and objects, gram-

ISSN 0953-4180/07/$17.00 © 2007 – IOS Press and the authors. All rights reserved matically correct speech, and preserved syntactic comprehension are present. A third variant, which differs from both NfPPA and SD is called "logopenic", and is characterized by a speech slowed in rate but without articulatory difficulties, syntactically simple but correct, halted by frequent word-finding pauses, in the absence of (syntactic or semantic) comprehension deficits [7].

Some reviews of demographic and clinical features of patients with diagnosis of NfPPA revealed that they are largely more males than females (66% vs 34%), and that the onset of the illness is shown between the age of 50 and 69, with the modal value at 64 years [23]. When the MRI shows abnormal findings, there are either in the left hemisphere or bilateral (for a review: Westbury, 1997 [23]).

We present here a new case of non-fluent primary progressive aphasia, characterized by right hemisphere involvement. The observation can be interpreted referring to the hypothesis of crossed aphasia (CAD), as originally defined by Bramwell [2]. He introduced this term to denote, in a broad sense, any aphasic syndrome resulting from a cerebral lesion ipsilateral to the dominant hand.

## 2. Case report

G.G. is a 56-years-old, right handed (50% dexterity, [1] following Biggs [3] evaluation), man. He used to work as a business manager and has had 13 years of education. His clinical history is unremarkable. Family history is positive for neurodegenerative diseases (grandfather and father with diagnosis of Alzheimer Disease).

By the age of 53$^{th}$, the patient presented difficulties in word-finding, as he referred at his first neurological consultation in 2004. This consultation showed a normal neurological examination, but MRI revealed aspecific and puntiform signal hyperintensity in the right frontal lobe and SPECT showed hypoperfusion in the right frontal cortex.

He was autonomous in his daily activities except for the ones requiring oral and written language production. Formal neuropsychological evaluation at that time revealed an isolated deficit on verbal fluency. The great difficulties in language expression, associated to an almost preserved comprehension, made the patient deeply depressed.

We reported G.G.'s neurological and neuropsychological evaluation, assessed three years after the onset of the symptomatology, investigating with particular care his language abilities.

### 2.1. Neurological evaluation

Neurological examination revealed the cranial nerves to be intact. The muscle tone was normal in the upper and lower limbs. Gait and reflexes were normal. Tremors at the distal extremities were evident.

### 2.2. General neuropsychology

Neuropsychological testing was administered by an experienced examiner in a quiet environment at the hospital.

Approximately 60 to 90 minutes were needed to administer tests. Language impairment prevented from long-term verbal memory evaluation that requires production of complex sentences (story recall [18]).

The examination included screening test for dementia (Mini-Mental State Examination, MMSE [6]), test of nonverbal reasoning (Raven's Coloured Progressive Matrices, CPM), auditory language comprehension (Token Test), verbal fluency with phonemic and semantic cues, verbal and spatial short-term memory (Digit Span forward and Spatial Span), spatial long-term memory (Rey Recall), constructional abilities (Rey's complex copy), upper limbs [4] and buccofacial apraxia [21], visuospatial abilities (VOSP – The Visual Object and Space Perception battery subtests of incomplete letter detection, object decision and number location detection [22], Judgement of lines orientation - H form [1]), and attention (Trail Making Test).

Table 1 reports GG's performance in these tasks.

In sum, G.G. showed deficits in all these cognitive domains, with the exception of short-term verbal memory, letter, object and number position detection, and buccofacial praxic abilities. In addition, language comprehension and non-verbal reasoning were at a borderline level.

### 2.3. Language assessment

G.G.'s spontaneous speech was non-fluent, with anomic pauses, phonetic substitutions and articulatory disturbances; oral language was made up of short sentences, of no more than 2–3 words; syntax was simple.

A formal language evaluation with Aachener Aphasia Test (Italian version [9]) and BADA [15] showed

[1] The degree of dexterity ranges from 0 to 100%, whereas left-handedness assumes negative values.

Table 1
General neuropsychology (July 2006)

| TEST | Original scores | Adjusted scores | Cut-off |
|---|---|---|---|
| MMSE (0–30) | 27 | 25.99 | 24 |
| Raven (CPM 0–36) | 18 | 18 | 18 |
| Rey Recall (0–36)* | 4 | 2.25 | 9.47 |
| Digit span | 5 | 4.75 | 3.75 |
| Spatial span* | 3 | 2.75 | 3.75 |
| Token test (0–36) | 30.5 | 28.5 | 26.5 |
| Fluency (phonological cue)* | 2 | 0 | 17 |
| Fluency (semantic cue)* | 0 | 0 | 25 |
| Rey copy (0–36)* | 20 | 19.75 | 28.88 |
| Trail making test A* | 97 | 91 | 93 |
| Trail making test B* | 392 | 376 | 282 |
| Trail making test B-A* | 295 | 285 | 186 |
| De Renzi right arm (0–72)* | 52 | | 62 |
| De Renzi left arm (0–72)* | 54 | | 62 |
| Buccofacial apraxia (0–20) | 19 | 18.75 | 17–18 |
| VOSP: | | | |
| Incomplete letters | 18 | | 16 |
| Object decision | 20 | | 14 |
| Number location | 8 | | 7 |
| Benton lines* | 11 | 14 | 17 |

*Score below cut-off.

difficulties repeating single words and sentences, associated with disorder of comprehension and verbs naming. Writing on composition was well performed, whereas hand writing, for both words and non-words was pathological and a qualitative analysis of his calligraphy showed an evident micrography.

Oral and written naming of verb appeared impaired. The nature of errors referred to the selection of wrong stem (i.e. "vote" rather than "put in envelope") or anomia (the pantomime of the gesture substitutes the verbs).

In comprehension BADA subtest, the patient demonstrated difficulties in tasks that require a judgment about sentence morphosyntactic features: subject – verb, noun – adjective and article – noun agreement, verb conjugation, location of constituents.

## 3. Discussion

In this report we described a case of NfPPA, a clinical syndrome due to a neurodegenerative process that affects primarily language functions. From the first clinical description of Mesulam [13], during recent years renewed interest has been developed in this form of aphasia that belongs to the spectrum of frontotemporal dementias (FTD).

The heritability of PPA is not well known. Krefft et al. [8] reported the first case of familial PPA, in which all members affected (three siblings of four) met criteria of PPA: genetic analysis revealed a probable autosomal inheritance (dominant or recessive).

Most studies in literature focused on neuropsychological and neuropathological characterisation of different forms of PPA, with particular concern of anatomical localization of brain damage. Despite the large data corpus, there is not a general consensus about brain areas involved in NfPPA, mainly for what concerns the lateralization of cerebral damage. Left brain involvement, consistently with the usual localization of language function, is well documented in several studies; among these the following imaging data may be mentioned: atrophy of the pars opercularis and triangularis of the left inferior frontal gyrus and grey matter loss in the left precentral gyrus of the insula [7]; cortical atrophy in inferior, orbital, insular, and dorsolateral regions of the left frontal lobe [11]; focal abnormality of the left insular region and frontal operculum extending onto the inferior frontal gyrus, with the epicentre of this hypometabolic cluster being in the anterior insula [17].

Bilateral anomalies were found in several cases, varying from 43% with MRI to 31% with PET or SPECT in Westbury's review [20,23].

The opposite pattern, with right side damage, is reported in left-handed patients by Mesulam [12] and Drezga [5].

Up to date and excluding our observation, only one case of right-handed subject showing right hypoperfusion at SPECT scan is reported in literature [19].

Table 2
Language assessment (July 2006)

| *Aachener Aphasie Test* | | | | |
|---|---|---|---|---|
| | *Score* | *PT* | *Gravity* | *Performance* |
| Repetition | 116/150 | 51 | 5 | Pathological |
| Written language | 82/90 | 64 | 8 | Normal |
| Objects naming | 118/120 | 80 | 9 | Normal |
| Comprehension | 105/120 | 60 | 7 | Pathological |
| *B.A.D.A* | | *Cut-off* | | |
| Non-words repetition | 31/35 | 33 | | Pathological |
| Non-words reading | 42/45 | 43 | | Pathological |
| Non-words writing | 25/25 | 23 | | Normal |
| Oral lexical decision | 78/80 | 78 | | Borderline |
| Visual lexical decision | 77/80 | 76 | | Normal |
| Words repetition | 32/45 | 43 | | Pathological |
| Words reading | 77/92 | 90 | | Pathological |
| Words writing | 41/46 | 44 | | Pathological |
| Oral objects comprehension | 39/40 | 38 | | Normal |
| Visual objects comprehension | 40/40 | 38 | | Normal |
| Oral verbs comprehension | 19/20 | 18 | | Normal |
| Visual verbs comprehension | 18/20 | 18 | | Borderline |
| Oral objects naming | 29/30 | 28 | | Normal |
| Oral verbs naming | 25/28 | 26 | | Pathological |
| Written objects naming | 22/22 | 20 | | Normal |
| Written verbs naming | 17/22 | 20 | | Pathological |
| Naming based in definition | 15/16 | 14 | | Normal |
| Oral morphosyntactic comprehension | 44/48 | 46 | | Pathological |
| Visual morphosyntactic comprehension | 17/24 | 22 | | Pathological |
| Sentences repetition | 12/20 | 18 | | Pathological |
| Oral syntactic comprehension | 55/60 | 58 | | Pathological |
| Visual syntactic comprehension | 38/45 | 43 | | Pathological |

In this paper authors described a 65-years woman who presented gradual decrease in fluency with intact cognitive functions: her brain SPECT revealed $^{99m}$Tc-HMPAO uptake reduction into the right frontal, perisylvian and temporal lobe regions.

This unusual finding is explained by authors referring to the hypothesis that women brain has greater bilateral representations of language. This is not true for our male patient.

Our data show that NfPPA may arise not only from cortical abnormalities in the language-dominant left hemisphere but also from right hemisphere involvement: this observation could be explained also referring to the hypothesis of crossed aphasia. Recently, Mariën et al. [10], reviewing a corpus of 152 case reports of CAD following cerebrovascular lesions, proposed a set of criteria allowing a classification of CAD into unreliable, possible and reliable.

If we extend these criteria to other possible etiologies (i.e. neurodegenerative disease), we can find that G.G. could be classified as "reliable CAD": in fact he met all the five points requested to assign this label (evidence of aphasia; evidence of natural right handedness; lesions restricted to right hemisphere; absence of familial left handedness and no history of early brain damage or seizures).

In conclusion, G.G.' cognitive profile seems comparable with a clinical picture of NfPPA, with the peculiarity of right-hemisphere involvement suggesting the presence of CAD.
